# Relationship Between the Urine Flow Rate and Risk of Contrast-Induced Nephropathy After Emergent Percutaneous Coronary Intervention

**DOI:** 10.1097/MD.0000000000002258

**Published:** 2015-12-18

**Authors:** Yong Liu, Lixia Lin, Yun Li, Hualong Li, Deng-Xuan Wu, Jian-bin Zhao, Dan Lian, Yingling Zhou, Yuanhui Liu, Piao Ye, Peng Ran, Chongyang Duan, Shiqun Chen, Pingyan Chen, Ying Xian, Jiyan Chen, Ning Tan

**Affiliations:** From the Department of Cardiology, Guangdong Cardiovascular Institute, Guangdong Provincial Key Laboratory of Coronary Disease, Guangdong General Hospital, Guangdong Academy of Medical Sciences, School of Medicine, South China University of Technology, Guangzhou, China (YL, LL, YL, HL, D-XW, J-BZ, DL, YZ, YL, PY, PR, SC, JC, NT); National Clinical Research Center for Kidney Disease, State Key Laboratory of Organ Failure Research, Department of Biostatistics, School of Public Health and Tropical Medicine, Southern Medical University, Guangzhou, China (CD, SC, PC); Department of Biostatistics, South China College of Cardiovascular Research, Guangdong Society of Interventional Cardiology, Guangzhou, China (SC); Duke Clinical Research Institute, Durham, North Carolina, USA (YX).

## Abstract

A low urine flow rate is a marker of acute kidney injury. However, it is unclear whether a high urine flow rate is associated with a reduced risk of contrast-induced nephropathy (CIN) in high-risk patients.

We conducted this study to evaluate the predictive value of the urine flow rate for the risk of CIN following emergent percutaneous coronary intervention (PCI).

We prospectively examined 308 patients undergoing emergent PCI who provided consent. The predictive value of the 24-hour postprocedural urine flow rate, adjusted by weight (UR/W, mL/kg/h) and divided into quartiles, for the risk of CIN was assessed using multivariate logistic regression analysis.

The cumulative incidence of CIN was 24.4%. In particular, CIN was observed in 29.5%, 19.5%, 16.7%, and 32.0% of cases in the UR/W quartile (Q)-1 (≤0.94 mL/kg/h), Q2 (0.94–1.30 mL/kg/h), Q3 (1.30–1.71 mL/kg/h), and Q4 (≥1.71 mL/kg/h), respectively. Moreover, in-hospital death was noted in 7.7%, 3.9%, 5.1%, and 5.3% of patients in Q1, Q2, Q3, and Q4, respectively. After adjusting for potential confounding predictors, multivariate analysis indicated that compared with the moderate urine flow rate quartiles (Q2 + Q3), a high urine flow rate (Q4) (odds ratio [OR], 2.69; 95% confidence interval [CI], 1.27–5.68; *P* = 0.010) and low urine flow rate (Q1) (OR, 2.23; 95% CI, 1.03–4.82; *P* = 0.041) were significantly associated with an increased risk of CIN. Moreover, a moderate urine flow rate (0.94–1.71 mL/kg/h) was significantly associated with a decreased risk of mortality.

Our data suggest that higher and lower urine flow rates were significantly associated with an increased risk of CIN after emergent PCI, and a moderate urine flow rate (0.94–1.71 mL/kg/h) may be associated with a decreased risk of CIN with a good long-term prognosis after emergent PCI.

## INTRODUCTION

Contrast-induced nephropathy (CIN) is a common complication following contrast media administration. It is the third most common cause of acute renal failure in hospitalized patients and is associated with poor early and late outcomes.^1^ In particular, patients undergoing emergent percutaneous coronary intervention (PCI) are reportedly at a significantly increased risk of developing CIN.^2^

The Risk, Injury, Failure, Loss, End-Stage Kidney Disease and Acute Kidney Injury Network criteria use serum creatinine levels and urine output (<0.5 mL/kg/h) to diagnose acute kidney injury (AKI).^3^ Moreover, in patients with subarachnoid hemorrhage undergoing a contrast study, CIN can be defined as an increase in urine output by <0.5 mL/kg/h during a 6-hour block.^4^ Additionally, a high urine flow rate may reduce the incidence of contrast-induced (CI)-AKI via a more rapid transit of contrast medium through the kidneys, enhanced contrast dilution in the renal tubule, and other effects.^5,6^ Furthermore, Gu et al^5^ have indicated that a higher urine output (3942 mL vs. 3112 mL), which was induced by furosemide and recorded for 28 hours, can significantly reduce the risk of CIN (8.1% vs. 14.2%) compared to a lower urine output. Hence, a higher urine output is associated with a lower risk of CIN in conditions where aggressive hydration is ensured to prevent CIN. In addition, the maintenance of a urine flow rate of >150 mL/h in the first 24 hours in patients scheduled for elective coronary angiography, with or without intervention, can help achieve a modest reduction in the rates of renal failure (21.6% vs. 45.9%).^6^ The administration of adrenomedullin before contrast exposure may reduce the risk of CIN and cause significant decreases in the absolute change in daily urine output (−0.53 ± 0.1 vs. −1.46 ± 0.5 mL/100 g body weight; *P* < 0.05).^7^

However, it is unclear whether a high postprocedural urine flow rate can reduce the incidence of CIN. In the present study, we aimed to evaluate the predictive value of the 24-hour postprocedural urine flow rate, adjusted by weight (UR/W, mL/kg/h), for the risk of CIN and long-term prognosis in high-risk patients undergoing emergent PCI.

## METHODS

### Subjects

In this prospective observational study, we examined 536 consecutive patients aged ≥18 years between March 2012 and December 2013 who agreed to stay in the hospital for 2 to 3 days after emergent coronary angiography, according to the institution's protocol. In accordance with the updated European Society of Urogenital Radiology Contrast Media Safety Committee guidelines,^8^ the exclusion criteria were pregnancy, lactation, intravascular administration of contrast medium within the last 7 or 3 days postoperatively (n = 46), lack of use of low-osmolarity contrast agents (n = 20), lack of emergent PCI (n = 12), missing postoperative urine volume records (n = 8), cardiovascular surgery or endovascular repair (n = 2), end-stage renal disease or renal replacement (n = 2), missing preoperative or postoperative creatinine data (n = 127), malignancy (n = 0), lack of use of isotonic saline for hydration (n = 10), and missing weight data (n = 1).

Finally, 308 patients with ST-elevation–myocardial infarction (STEMI) or those with non-ST-elevation acute coronary syndromes (NSTE-ACS) who were very high risk (ie, those with refractory angina, severe heart failure, life-threatening ventricular arrhythmias, or hemodynamic instability) and underwent emergent PCI were included in the analysis.^9^ Follow-up events were carefully monitored and recorded by trained nurses through office visits and telephone interviews at 1, 6, 12, and 24 months after coronary angiography. The mean follow-up time was 1.73 ± 0.35 years (median, 1.77 years; interquartile range, 1.46–1.99 years).

The institutional Ethics Research Committee approved the study, and all patients provided written informed consent.

### Emergent PCI

Emergent PCI was defined as primary PCI for patients with STEMI and immediate PCI (ie, <2 hours from hospital admission, analogous to STEMI management) for patients with NSTE-ACS who were very high risk.^9^ Based on the lesions and the patients’ other conditions, our interventional team used general guiding catheter, guiding wires, balloon catheters, and stents via the right femoral or radial access according to the individuals’ experiences and clinical guideline.^9^ The contrast volume and types (nonionic, low-osmolarity [either Iopamiron or Ultravist], both 370 mg I/mL) were left to the interventional cardiologist's discretion and depended on the patient's condition. Patients were treated according to the European Society of Cardiology and American College of Cardiology Foundation/American Heart Association guidelines.^9,10^ Serum creatinine concentrations were collected in all patients on admission and 3 days postprocedure according to the local institutional protocol.^11^

The creatinine clearance (CrCl) values were calculated by applying the Cockcroft–Gault formula using the measured serum creatinine concentration.^12^ According to the 2010 European Society of Urogenital Radiology Contrast Media Safety Committee guidelines,^8^ all patients received a continuous intravenous infusion of isotonic saline at a rate of 1 mL/kg/h (.5 mL/kg/h in cases of left ventricular ejection fraction <40% or severe congestive heart failure) at the start of the procedure or just before the procedure, which was continued from 4 to 24 hours before the procedure to 6 to 24 hours after the procedure. The urine volume within 24 hours after the procedure (mL) and the patients’ weight (kg) were recorded, and the urine output rate to weight ratio (UR/W, mL/kg/h) was calculated.

### Study Endpoints

The primary endpoint was CIN_25_, defined as an increase in the serum creatinine concentration by ≥25% or ≥0.5 mg/dL compared to the baseline value within 72 hours of contrast exposure.^13^ We also followed and recorded the in-hospital clinical outcomes, including renal replacement therapy, acute heart failure, recurring acute myocardial infarction, intraaortic balloon pump (IABP) use, arrhythmia, stroke, bleeding, and death, as well as long-term major adverse clinical events (MACE) such as death, nonfatal myocardial infarction, target vessel revascularization, CIN requiring renal replacement therapy, stroke, and rehospitalization.

### Statistical Analysis

For continuous variables, 1-way analysis of variance was performed for normally distributed data (expressed as mean ± standard deviation), whereas the Kruskal–Wallis test was used for nonnormal distributions (presented as median and interquartile range). Pearson Chi-square test or Fisher exact test was used, as appropriate, for categorical data, which were expressed as percentages. The odds ratios (ORs) of CIN for subgroups with different UR/W ratios (cutoff values determined based on the quartiles) were estimated in unadjusted and adjusted stepwise logistic regression analyses; collinear variables were not retained in the final model. After a trade-off between overfitting and appropriate control of unbalanced factors, we finally used the factors with *P* values < 0.05 at the baseline analysis, along with clinically important factors, for multivariate logistic regression analysis. Reanalysis of all the models, after excluding factors for which considerable data were missing (eg, left ventricular ejection fraction [LVEF] was missing from >40 cases), was also attempted. Moreover, the Kaplan–Meier method and log-rank test were used to compare mortality and MACE rates according to the UR/W quartiles. Multivariate Cox regression analyses, adjusted for a CrCl of <60 mL/min, were performed. Only the available rates were assessed, and cases with missing data were excluded. All data analyses were performed using SAS, version 9.4 (SAS Institute, Cary, NC) and R software (version 3.1.2, R Foundation for Statistical Computing, Vienna, Austria).^14^ A 2-sided *P*-value < 0.05 was considered statistically significant.

## RESULTS

### Baseline Characteristics

The mean age of the 308 patients (236 men, 76.6%) was 61 ± 12 years, and the patients were stratified into UR/W quartiles: ≤0.94 mL/kg h, 0.94–1.30 mL/kg h, 1.30–1.71 mL/kg h, and ≥1.71 mL/kg h. Tables 1 and 2 show the baseline clinical, biochemical, and angiographic parameters according to the UR/W quartiles. There were no intergroup differences in terms of sex, age, the LVEF, congestive heart failure, use of an IABP, the Mehran risk score, chronic kidney disease (CKD), anemia, diabetes mellitus, a previous history of coronary artery bypass grafting, and MI, as well as procedural characteristics such as the contrast volume, number of stents, and procedure duration. However, there were intergroup differences in terms of the 24-hour postprocedural total hydration rate to weight ratio (HR/W, mL/kg/h), which included intravenous and oral hydration.

**TABLE 1:**
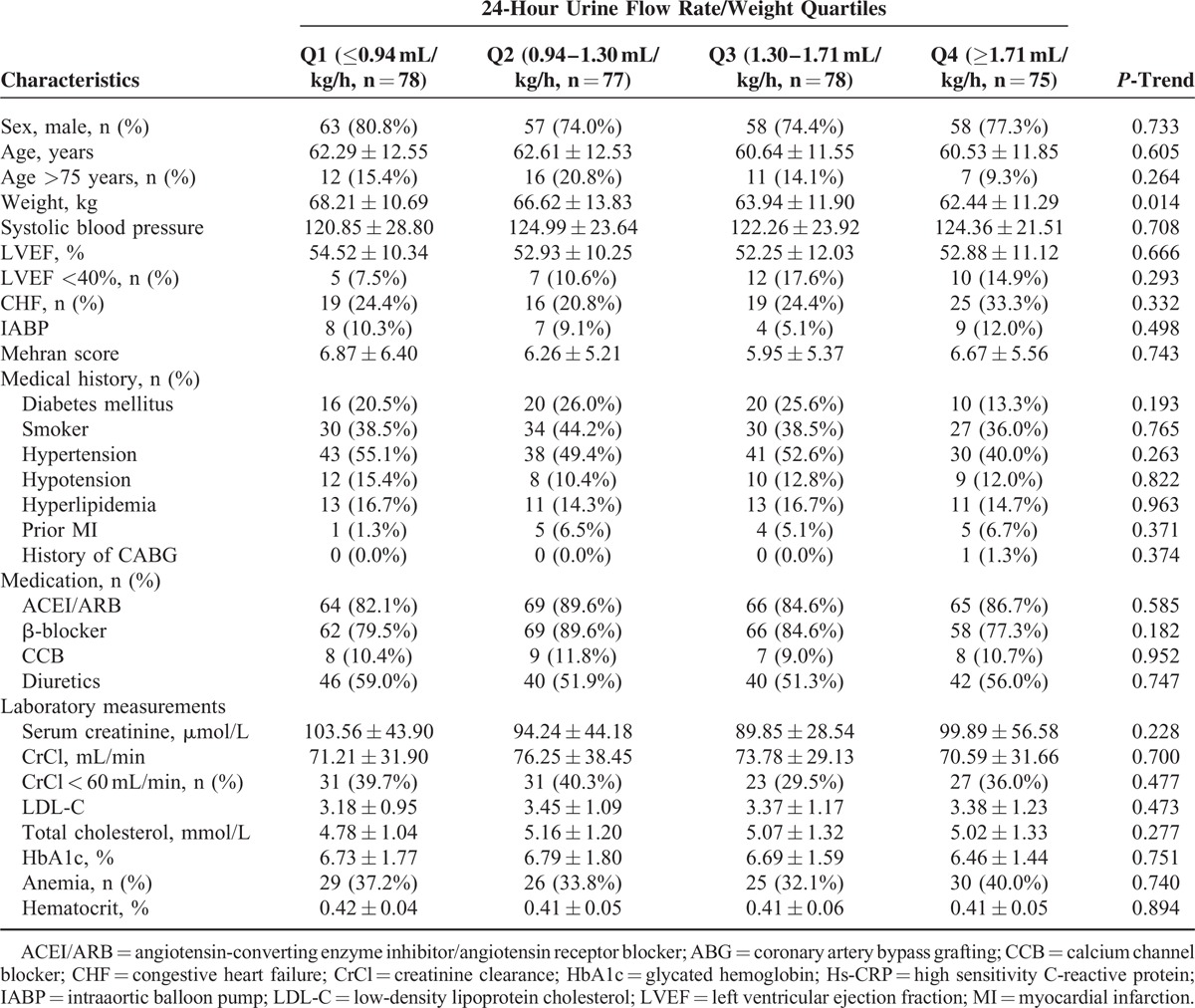
Baseline Patient Characteristics According to the 24-Urine Flow Rate Quartiles

**TABLE 2:**
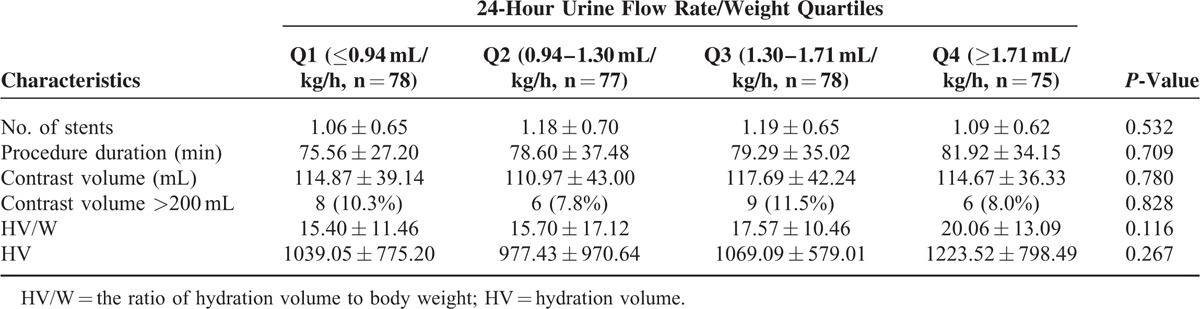
Baseline Procedural Characteristics of the 24-Hour Urine Flow Rate Quartiles

### Urine Flow Rate for Predicting CIN

The incidence of CIN and in-hospital clinical outcomes according to the UR/W quartiles is shown in Table 3. To investigate the association between the urine flow rate and CIN, multivariate stepwise logistic regression analysis was performed (Table 4). After adjusting for age, female sex, CrCl, LVEF <40%, use of an IABP, HR/W, contrast volume, and use of diuretics and an angiotensin-converting enzyme inhibitor/angiotensin receptor blocker, multivariate stepwise logistic regression analysis indicated that compared with the moderate urine flow rate quartiles (Q2 and Q3), a high urine flow rate (Q4) (OR, 2.51; 95% confidence interval [CI], 1.23–5.14; *P* = 0.012) and low urine flow rate (Q1) (OR, 2.14; 95% CI, 1.02–4.51; *P* = 0.044) were significantly associated with an increased risk of CIN (Table 4). The independent predictors of CIN included female sex (OR, 2.46; *P* = 0.006), LVEF < 40% (OR, 2.84; *P* = 0.010), and diuretic use (OR, 2.44; *P* = 0.006).

**TABLE 3:**
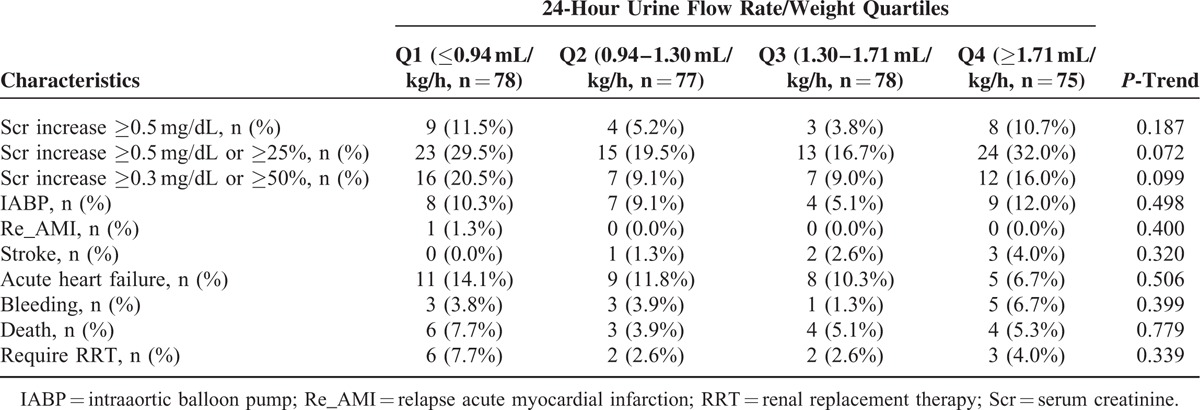
Incidence of Contrast-Induced Nephropathy and In-Hospital Clinical Outcomes According to the 24-Urine Flow Rate Quartiles

**TABLE 4:**
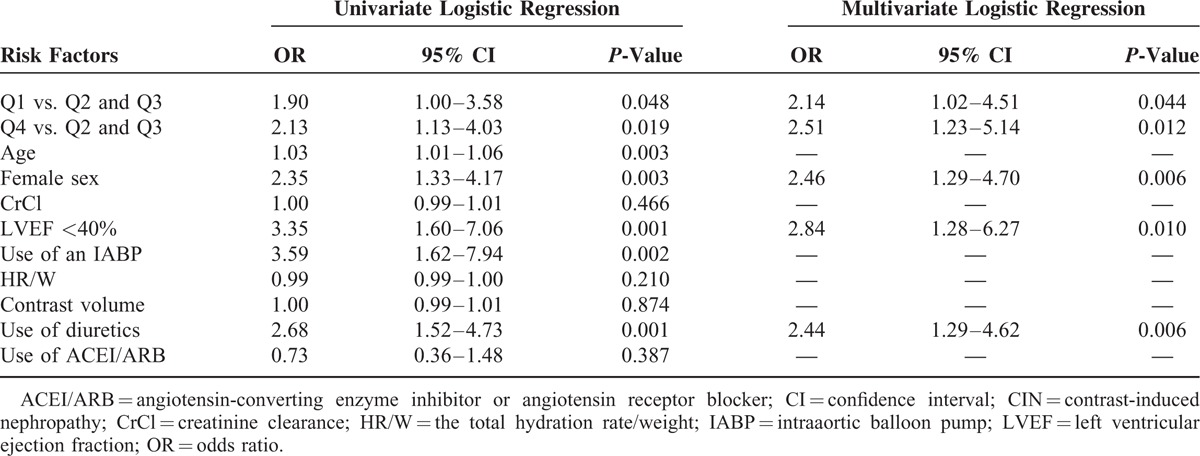
Univariate Analyses and Multivariate Associations Between CIN25 and the 24-h Urine Flow Rate Quartiles

### Follow-Up

**FIGURE 1:**
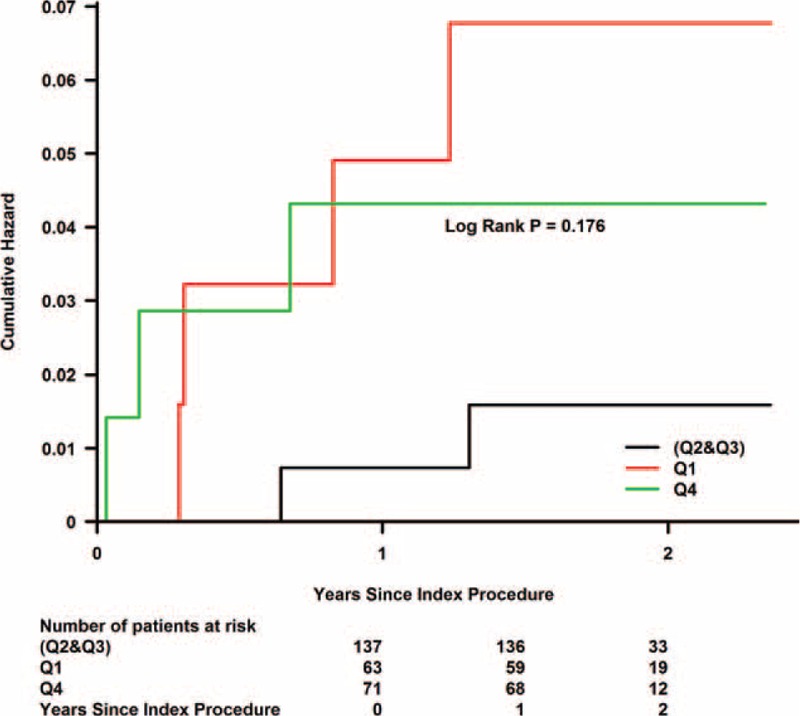
Kaplan–Meier curves showing the cumulative probability of mortality. Q1 = first quartile, Q2 and Q3 = second quartile and third quartile, Q4 = fourth quartile.

**FIGURE 2:**
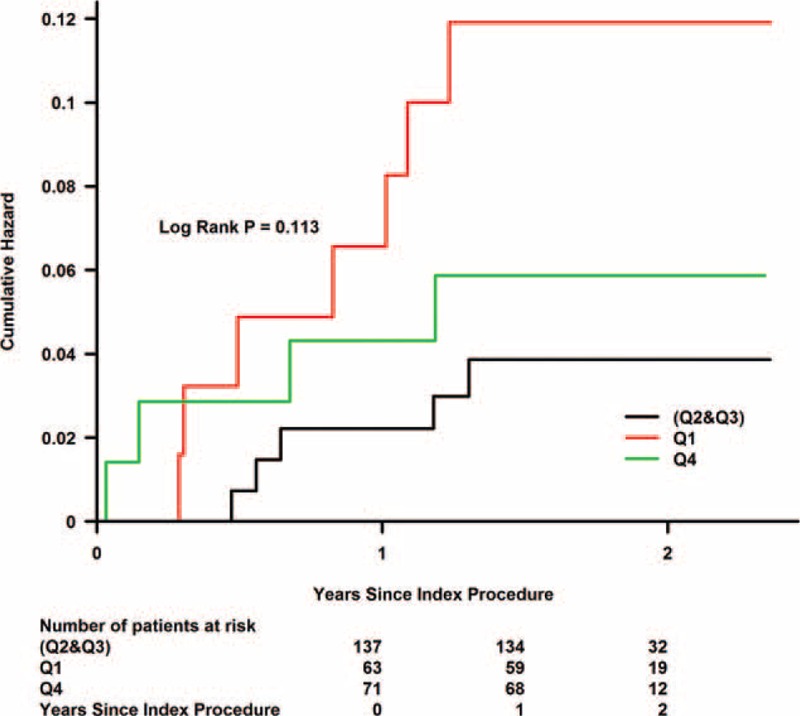
Kaplan–Meier curves showing the cumulative probability of major adverse clinical events. Q1 = first quartile, Q2 and Q3 = second quartile and third quartile, Q4 = fourth quartile.

During a median follow-up of 1.77 years (interquartile range, 1.46–1.99 years), Kaplan–Meier curve analyses showed that the risk of death and MACE during follow-up increased in the highest and lowest urine flow rate quartiles (Figs. 1 and 2).

**FIGURE 3:**
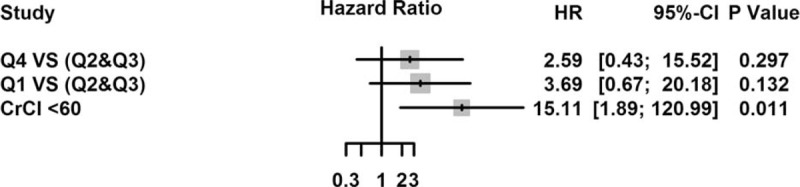
Adjusted hazard ratios of the Cox analysis for mortality. Q1 = first quartile, Q2 = second quartile, Q3 = third quartile, Q4 = fourth quartile, CrCl = creatinine clearance.

**FIGURE 4:**
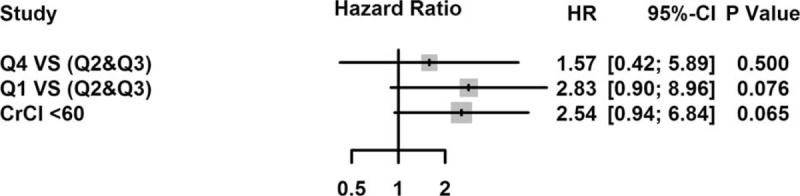
Adjusted hazard ratios of the Cox analysis for major adverse clinical events. Q1 = first quartile, Q2 = second quartile, Q3 = third quartile, Q4 = fourth quartile, CrCl = creatinine clearance.

A Cox regression model, including CrCl < 60 mL/min as a variable, indicated that patients in the second and third urine flow rate quartiles had a relatively lower risk of subsequent death (Q4 vs. Q2 and Q3: HR, 2.59; 95% CI, 0.43–15.52; *P* = 0.297; Q1 vs. Q2 and Q3: HR, 3.69; 95% CI, 0.67–20.18; *P* = 0.132) and lower MACE rates (Q4 vs. Q2 and Q3: HR, 1.57; 95% CI, 0.42–5.89; *P* = 0.500; Q1 vs. Q2 and Q3: HR, 2.83; 95% CI, 0.90–8.96; *P* = 0.076) than the fourth and first urine flow rate quartiles (Figs. 3 and 4).

## DISCUSSION

In the present study, we evaluated the predictive value of the urine flow rate for the risk of CIN after emergent PCI. Our data suggested that high and low urine flow rates are significantly associated with an increased risk of CIN after emergent PCI.

The risk of CIN in the present study was as high as that reported in recent studies on patients undergoing emergent or primary PCI.^2,15^ Adequate parenteral volume repletion is the cornerstone for CIN prevention, as hydration can increase urine flow rates, reduce the concentration of contrast media in the tubule, and expedite the excretion of contrast media, thus reducing the length of time that the tubular cells are exposed to the toxic effects of contrast media.^16,17^ Moreover, iodinated contrast media may induce hyperadrenergia by affecting thyroid function and the chemoreceptor response to metabolic acidosis, and thus may diminish urine output.^18^ Furthermore, a high urine flow rate may reduce the incidence of CI-AKI via a combination of physiological effects, including a more rapid transit of contrast medium through the kidneys, enhanced contrast dilution in the renal tubule (leading to the potential reduction of oxygen consumption in the medulla), and maintenance of flow in the renal tubules and collecting ducts, which may reduce the precipitation of contrast media in tubular cells.^5,6,19^

We observed that when the urine flow rate is maintained within a suitable range, a higher 24-hour urine flow rate is significantly associated with a lower risk of CIN in patients undergoing emergent PCI. Similarly, the PRINCE study showed that a high urine flow rate (≥150 mL/h) in the first 24 hours after the procedure can reduce the incidence of CI-AKI in patients who have undergone elective coronary angiography (21.6% vs. 45.9%); moreover, patients with a urine flow rate ≥150 mL/h developed acute renal failure but did not need dialysis.^6^ McCullough and Manley^20^ also suggested that adequate prehydration and maintenance of post-PCI urine flow rates at >150 mL/min are the most prudent measures.

However, Majumdar et al^21^ found that forced euvolemic diuresis led to a significantly increased risk of CIN in patients with CKD, even though a high urine output was achieved. In addition, a recent study showed that the incidence of CIN remains significantly high in patients receiving diuretics, whereas the urine output was not significantly different between the patients receiving diuretics and those receiving saline for 24 hours after angiography.^22^ Thus, regardless of whether a high output is achieved, the administration of diuretics has an effect on the incidence of CIN. A previous study demonstrated that a significantly higher urine output for 24 hours, induced by low-dose furosemide administration, was associated with a significantly lower incidence of CIN (8.1% vs. 14.2%) than that in a control group,^5^ which is consistent with our finding that diuretic use is an independent predictor of CIN; hence, this parameter was adjusted in the multivariate logistic regression analysis to exclude its effect in the present study. However, the results from the aforementioned study may also be related to the patients’ baseline characteristics and fluid balance.

In the present study, the presence of a urine flow rate within the moderate range was associated with a significantly lower risk of CIN in patients undergoing emergent PCI. This association may be related to the fluid balance. The RenalGuard system was developed for optimal hydration therapy, and it ensures a high urine output while simultaneously balancing the urine output and venous fluid infusion to prevent CIN. A previous study indicated that CIN occurred in 11% of patients in the RenalGuard group and in 20.5% of those in the control group (*P* = 0.025).^23^ The MYTHOS trial indicated that by administering low-dose furosemide, maintaining the intravascular volume, and minimizing the risk of overhydration or underhydration in patients with CKD, 4.6% of patients who received furosemide with a matched hydration strategy developed CIN compared to 18% in the hydration group (*P* = 0.005).^24^ However, we did not have sufficient evidence to prove that the patients with high or low urine flow rates suffer from fluid imbalance.

The relationship between postprocedural urine output and CIN (after emergent PCI) or clinical outcomes remains unclear. Hence, further randomized studies are essential.

## LIMITATIONS

The present study has certain limitations. First, as this prospective observation study was conducted at a single center, the evidence may not be as strong as that obtained from a randomized controlled trial. Second, this study included a relatively small sample size without enough power to make a conclusion, as it was a pilot prospective observation study without precise calculation of a sample size; thus, we will conduct another larger multicenter study to further verify our findings in the future. Third, the CrCl was computed using the Cockcroft–Gault formula rather than by direct measurement. Fourth, the variation in measurement times may have led to missed peak levels of creatinine after the procedure. Fifth, both the creatinine and urine flow rate 5 to 7 days after emergent PCI were missing. The lack of creatinine data may have led to underestimating the true incidence of CIN in the present study population. Finally, the confounding effect of a fluid imbalance on CIN prevention existed in this study; however, after adjusting for HR/W, the highest urine flow rate and lowest urine flow rate were still significantly associated with an increased risk of CIN compared with the moderate urine flow rate.

## CONCLUSIONS

We found that a higher 24-hour urine flow rate, in the range of 0.94 to 1.71 mL/kg/h, is associated with a significantly lower risk of CIN in patients who underwent emergent PCI; however, a urine flow rate exceeding this value may lead to the opposite effect, as well as a lower urine flow rate.
